# Angiographic evaluation of radial artery injury after transradial approach for percutaneous coronary intervention

**DOI:** 10.1007/s12928-020-00750-7

**Published:** 2021-02-26

**Authors:** Takenori Kanazawa, Kiyotaka Shimamura, Kazuya Nagao, Hiroshi Yukawa, Kenji Aida, Yohei Kobayashi, Naoki Takahashi, Eiichiro Nakagawa, Haruyasu Itoh, Fujio Hayashi, Toshinori Makita, Masaru Tanaka, Tsukasa Inada

**Affiliations:** Department of Cardiology, Osaka Red Cross Hospital, 5-30 Fudegasakicho, Tennoji-ku, Osaka, 543-8555 Japan

**Keywords:** Radial artery angiography, Radial artery occlusion, Radial artery stenosis

## Abstract

The transradial approach for percutaneous coronary intervention (TRA-PCI) has been increasingly gaining popularity in clinical practice. However, its association with risk for long-term radial artery injury has not been yet thoroughly defined. We retrospectively examined the patients undergoing radial artery angiography (RAG) after TRA-PCI to determine the incidence and risk factors of radial artery injury. The study included 558 patients undergoing follow-up radial artery angiography at 12 month after TRA-PCI. Radial artery injury occurred in 140 patients (25%) with 3 distinct morphological patterns: focal radial artery stenosis (RAS) P.7,7: in 7 patients (1%), diffuse radial artery stenosis (RAS) in 78 patients (14%), and radial artery occlusion (RAO) in 55 patients (10%). Patients with RAS/RAO were more likely to be female, had smaller height and body weight, smaller body mass index and smaller body surface area (BSA) as compared with those without RAS/RAO. Multivariable logistic regression analysis identified BSA (odds ratio, 1.34 per 0.1 m^2^ increase; 95% confidence interval, 1.07–1.71; *p* = 0.01) and a history of TRA-PCI (odds ratio, 2.35; 95% confidence interval, 1.16–5.08; *p* = 0.017) as independent predisposing factors of radial artery injury. In a sub-analysis of 323 patients undergoing both pre-PCI RAG and follow-up RAG, pre-PCI radial diameter as well as BSA and a history of TRA-PCI were independently associated with radial artery injury. Long-term injury after TRA-PCI is considerably common and care should be paid for RAS/RAO, especially for those patients with lower BSA, history of TRA-PCI and small radial artery diameter.

## Introduction

In the field of percutaneous coronary intervention (PCI), the transradial approach (TRA) has taken the place of the transfemoral approach and has been widely used worldwide [1–3], Reportedly, TRA-PCI is associated with a shorter hospital stay and lower incidences of mortality and adverse cardiac events [4–7]. TRA-PCI, however, can cause radial artery injury, which can result in radial artery stenosis (RAS) or radial artery occlusion (RAO). Although radial artery injury is a quiescent complication and often overlooked, it can be a serious problem when using the radial artery as an access site for repeated TRA-PCI or as a conduit for coronary artery bypass graft or hemodialysis fistula formation [8, 9]. Previously reported incidences of RAO varied widely across the studies from 1 to 33% [10]. Most of these studies evaluated RAO using ultrasound at early phase after TRA-PCI. However, ultrasound might not fully capture the morphological patterns of RAS/RAO, and radial artery angiography (RAG) might be more precise for the anatomical assessment of radial artery [11]. In addition, there have been few reports evaluating long-term injury of radial artery after TRA-PCI [12, 13]. Therefore, in the present study, we angiographically evaluated the patterns, incidences and predisposing factors of 12-month radial injury after TRA-PCI using RAG.

## Methods

### Study design

This study enrolled patients undergoing follow-up RAG after TRA-PCI at our hospital from January 2004 to October 2014. During the study period, in accordance with our institutional protocol, we mainly used the right brachial artery as an access site for coronary angiography (CAG) unless patients were receiving hemodialysis or scheduled for ad hoc PCI. When stenotic lesions requiring PCI were detected by CAG (pre-PCI CAG), we performed RAG via the arterial sheath inserted in the brachial artery to ascertain whether radial artery patency was adequate for TRA-PCI. We usually performed TRA-PCI within 1 month after CAG. All patients received antiplatelet therapy with aspirin (≥ 81 mg/day) and clopidogrel (75 mg/day); drug-eluting stents were implanted in most patients. Patients were encouraged to undergo 12-month follow-up CAG unless they had severe renal dysfunction. At follow-up CAG, we used the brachial artery of the same side that we had used at pre-PCI CAG. Regarding patients undergoing TRA-PCI without RAG before PCI, such as those undergoing ad hoc TRA-PCI, we performed RAG at follow-up CAG unless the access site at follow-up CAG was different from TRA-PCI. Regarding patients undergoing TRA-PCI multiple times during the study period, only the outcome of follow-up RAG after the index TRA-PCI was included in this study.

### Ethics

This study was performed in accordance with the provisions of the Declaration of Helsinki and the guidelines for epidemiological studies issued by the Ministry of Health, Labour, and Welfare of Japan. Informed consent from patients participating in the study was waived because of the retrospective design. This study was approved by the institutional review board.

### CAG and RAG procedures

All patients underwent right brachial artery puncture after local anesthesia with xylocaine 1%. To avoid complications of puncture site reported in the previous studies using 6-Fr sheath [14], we used a 5-Fr arterial sheath (Radifocus Introducer II; Terumo, Tokyo, Japan) for both pre-PCI and follow-up diagnostic catheterization. After successful insertion of a 5-Fr brachial arterial sheath, CAG and left ventriculography were performed. At the end of the catheterization, RAG was performed by injecting contrast agents directly into the arterial sheath. To prevent a vasospastic response of the radial artery caused by contrast agents, we administered 1.0 mg isosorbide dinitrate via the arterial sheath immediately before RAG.

### TRA-PCI procedure

We used a 6-Fr arterial sheath (Radifocus Introducer II; Terumo) for TRA-PCI. All patients underwent right radial artery puncture after local anesthesia with xylocaine 1%. After successful insertion of a radial arterial sheath, we administrated 0.5 mg isosorbide dinitrate via the arterial sheath to prevent radial artery spasm. Heparin (100 IU/kg) was also administered via the sheath immediately after administering isosorbide dinitrate; additional heparin was administered in hourly boluses throughout the procedure. At the end of the TRA-PCI procedure, 0.5 mg isosorbide dinitrate was injected via the sheath followed by the removal of the arterial sheath and compression of the puncture site. We routinely used a compression device (TR Band: Terumo) to achieve hemostasis. Per the device protocol, we compressed the puncture site by injecting 13 ml of air into the cuff applied over the puncture site while simultaneously removing the sheath. After remaining the band in place for 2 h, we removed 2 ml of the cuff air every 1 h. After 6 h of compression, we completely removed air from the band. After confirming complete hemostasis, we unfastened and removed the band. If bleeding occurred during the compression, we injected additional 2 ml of air until bleeding would stop and extended each step by at least 1 h. Therefore, for the patients with bleeding, total duration of compression was extended beyond 6 h until complete hemostasis was achieved.

### Measurement of radial artery diameter

In patients undergoing pre-PCI RAG, we estimated the radial artery diameter using the diameter of the 5-Fr sheath as a relative value. First, we measured the diameter of the 5-Fr sheath in the radial artery at 5 cm upper from the wrist joint. Then, we calculated the radial artery diameter by multiplying the obtained sheath diameter and 1.91 mm, the absolute diameter of the 5-Fr sheath.

### Angiographic definitions of RAS and RAO

**Fig. 1:**
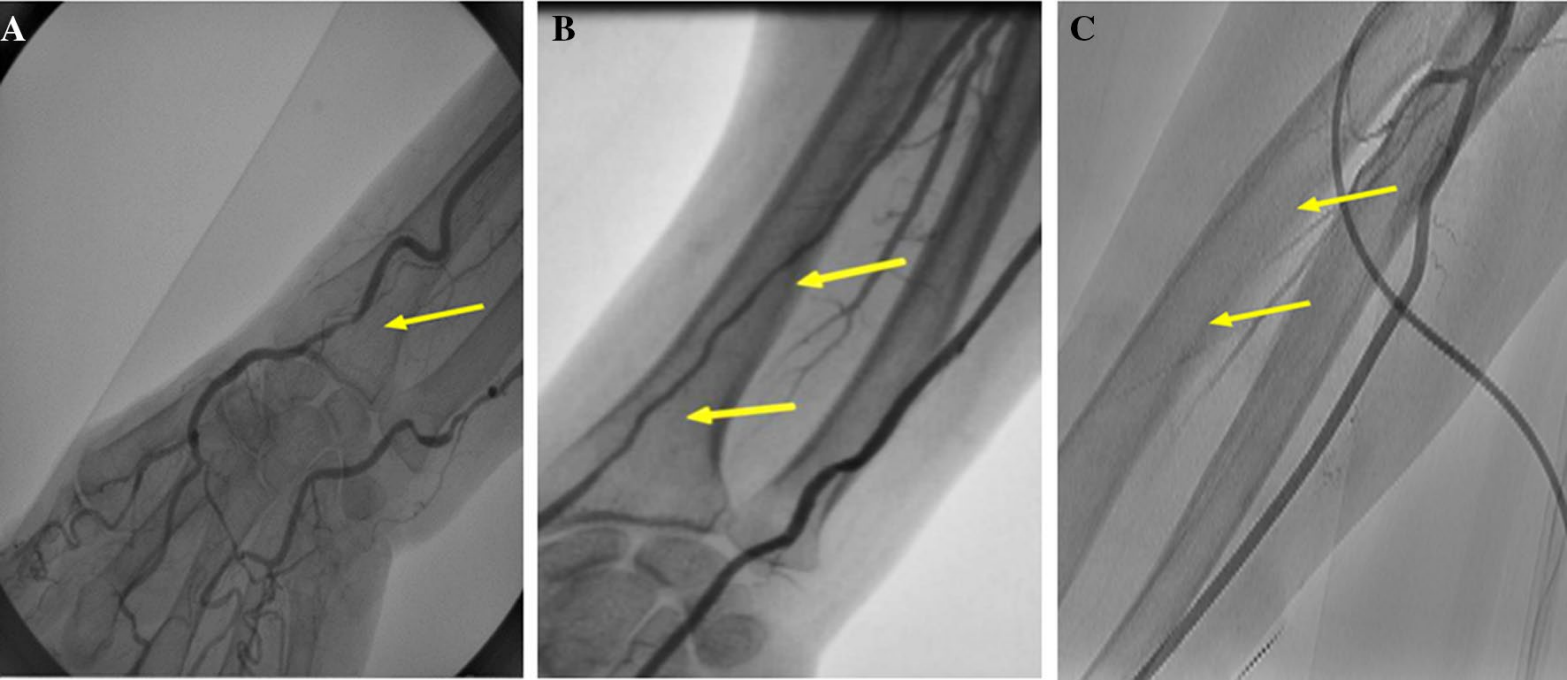
Morphological patterns of radial artery injury were defined as follows: focal radial artery stenosis as percent diameter stenosis > 50% and the length of the stenotic lesion < 10 mm (**a**); diffuse radial artery stenosis as percent diameter stenosis > 50% and the length of the stenotic lesion ≥ 10 mm (**b**); and radial artery occlusion as total radial artery occlusion (**c**). The arrows indicate stenosis or occlusion of the radial artery

We defined RAS and RAO on the basis of radial artery angiograms as follows: RAS as percent diameter stenosis > 50% and RAO as total radial artery occlusion. Additionally, we classified RAS into two types: RAS with the length of the stenotic lesion < 10 mm (focal RAS) and that with the length of the stenotic lesion ≥ 10 mm (diffuse RAS) (Fig. 1).

### Statistical analysis

Continuous variables are expressed as mean ± standard deviation or numbers with relative percentages as appropriate. Differences between patients with and without RAS/RAO were analyzed using the *t* test and the chi-square test. Variables independently associated with RAS/RAO were identified by a multivariable logistic regression analysis, and clinically relevant factors were incorporated into both the univariate and multivariable logistic regression analyses. In a sub-analysis of patients undergoing both pre-PCI and follow-up RAG, we constructed a parsimonious model with the 8 clinically most relevant variables because of a small number of patients with outcome. In this model, we included pre-PCI radial artery diameter as a potentially explanatory variable. A *p* value of < 0.05 was considered statistically significant. JMP 12.0.1 (SAS Institute, Cary, NC, USA) was used for all statistical calculations.

## Results

### Patient characteristics

Among the entire cohort of 558 patients undergoing follow-up RAG, 140 patients (25%) had radial artery injury at follow-up RAG. Their morphological patterns were focal RAS in 7 patients (1%), diffuse RAS in 78 patients (14%), and RAO in 55 patients (10%) (Fig. 1 and Table 1). Patients with RAS/RAO were more likely to be female, had smaller height and body weight, smaller body mass index and smaller body surface area (BSA) as compared with those without RAS/RAO. The proportion of patients with a history of PCI, hypertension, dyslipidemia, diabetes, acute coronary syndrome and a use of anticoagulants was not significantly different between patients with and without RAS/RAO. In addition, no significant difference between patients with and without RAS/RAO was observed in procedure time and proportion of patients requiring prolonged compression of puncture site due to bleeding. Patients with a history of TRA-PCI tended to be lower in patients with RAS/RAO than in those without (Table 1). None of the patients with RAS/RAO had anatomical variations of the radial artery including high origin of the radial artery, severe tortuosity, or radioulnar loop. We did not experience any complications on the cannulation to radial artery, but there were 4 patients (0.7%) who developed pseudo-aneurysm at the brachial access site which was treated surgically.

**Table 1:** Patient characteristics of the entire cohort

	Total (*N* = 558)	RAS/RAO (–) (*n* = 418, 75%)	RAS/RAO ( +)				*p* value for RAS/RAO (–) vs RAS/RAO ( +)
			Total (*n* = 140, 25%)	Focal RAS (*n* = 7, 1%)	Diffuse RAS (*n* = 78, 14%)	RAO (*n* = 55, 10%)	
Age, years	67.5 ± 9.5	67.1 ± 9.2	68.6 ± 10.2	71.4 ± 7.4	70.2 ± 9.7	66.1 ± 10.5	0.096
Women	116 (21)	72 (17)	44 (31)	3 (43)	26 (33)	15 (27)	0.007
Height, cm	161.4 ± 9.1	162.0 ± 8.9	159.5 ± 9.4	155.5 ± 7.9	159.7 ± 9.3	159.8 ± 9.4	0.0052
Body weight, kg	64.6 ± 12.6	65.9 ± 12.2	60.9 ± 13.1	57.0 ± 9.3	60.3 ± 13.1	62.3 ± 13.1	< 0.001
Body mass index, kg/m^2^	24.7 ± 3.7	25.0 ± 3.6	23.7 ± 3.6	23.7 ± 4.6	23.4 ± 3.6	24.2 ± 3.5	0.0003
Body surface area, m^2^	1.72 ± 0.21	1.74 ± 0.21	1.6 6 ± 0.21	1.58 ± 0.14	1.65 ± 0.20	1.68 ± 0.22	< 0.001
History of smoking	291 (52)	219 (52)	72 (51)	1 (14)	40 (51)	31 (56)	0.85
History of PCI	208 (37)	154 (37)	54 (39)	2 (29)	29 (37)	23 (42)	0.76
History of TRA-PCI	74 (13)	62 (15)	12 (9)	0 (0)	10 (13)	2 (3.6)	0.062
Hypertension	312 (56)	236 (56)	78 (56)	4 (57)	43 (55)	30 (55)	0.84
Dyslipidemia	314 (56)	238 (57)	78 (55)	5 (71)	36 (46)	37 (67)	0.92
Diabetes	223 (40)	166 (40)	57 (41)	4 (57)	31 (40)	22 (40)	0.84
Acute coronary syndrome	68 (13)	53 (13)	15 (11)	1 (14)	7 (9)	7 (13)	0.66
Procedure time (min)	53 ± 36	53 ± 36	52 ± 35	36 ± 8	55 ± 37	49 ± 33	0.38
Oral anticoagulants	49 (9)	37 (9)	12 (9)	0 (0)	6 (8)	6 (11)	0.92
Puncture site bleeding requiring prolonged compression	134 (24)	93 (22)	41 (29)	4 (6)	21 (27)	16 (29)	0.096

Data are n (%) or mean (SD), unless otherwise specified

*PCI* percutaneous coronary intervention, *RAS* radial artery stenosis, *TRA* transradial approach

### Factors related to RAS/RAO

In univariate analysis, women, height, body weight, body mass index, and BSA were related to RAS/RAO. In multivariable analysis, BSA and a history of TRA-PCI were independently associated with RAS/RAO (Table 2).

**Table 2:** Factors related to radial artery injury in the entire cohort

	Univariate analysis		Multivariable analysis	
	Odds ratio (95% confidence interval)	*p* value	Odds ratio (95% confidence interval)	*p* value
Age	0.98 (0.96–1.00)	0.96	1 (0.98–1.03)	0.94
Women	0.20 (0.06–0.69)	0.007	0.65 (0.35–1.21)	0.32
Body surface area (per log 1.1 m^2^ increase)	1.4 (1.19–1.64)	< 0.001	1.34 (1.07–1.71)	0.01
History of smoking	1.10 (0.35–1.47)	0.85	0.72 (0.46–1.12)	0.15
History of PCI	1.07 (0.73–1.60)	0.76	0.74 (0.47–1.17)	0.20
History of TRA-PCI	0.54 (0.28–1.03)	0.062	2.35 (1.16–5.08)	0.017
Hypertension	0.95 (0.65–1.40)	0.84	1.12 (0.74–1.71)	0.56
Dyslipidemia	0.97 (0.66–1.43)	0.92	0.90 (0.59–1.36)	0.62
Diabetes	1.04 (0.71–1.54)	0.84	0.87 (0.58–1.32)	0.51
Acute coronary syndrome	0.83 (0.45–1.52)	0.66	1.37 (0.73–2.72)	0.34
Procedure time	1.48 (0.29–8.91)	0.65	1.25 (0.23–7.80)	0.80
Oral anticoagulants	0.97 (0.49–1.90)	0.92	1.23 (0.62–2.61)	0.56
Puncture site bleeding requiring prolonged compression	1.44 (0.94–2.23)	0.09	0.79 (0.50–1.27)	0.33

Height, body weight, and body mass index were omitted from Table 3 due to possible multicollinearity

*PCI* percutaneous coronary intervention, *TRA* transradial approach

### Conversion of puncture site due to RAS/RAO at the repeated PCI

At follow-up catheterization, there were 60 patients who had both coronary artery stenosis on CAG requiring repeated PCI and RAS/RAO on RAG (RAS: 39 patients, RAO: 21 patients). Among those patients, access site for the subsequent repeated PCI was converted from the injured radial artery to other sites (i.e. the opposite radial artery or femoral artery) beforehand in 49 patients (82%): 28 in the RAS group and 21 in the RAO group.

### Analysis in the patients who underwent both pre-PCI RAG and follow-up RAG

Table 3 shows the characteristics of the 323 patients undergoing both pre-PCI RAG and follow-up RAG. In general, patient characteristics of this subpopulation were not largely different from those of the entire cohort, except for a lower proportion of patients with acute coronary syndrome. No patients had RAS/RAO on pre-PCI RAG, while 84 patients (26%) had radial artery injury at follow-up RAG: focal RAS, 3 patients (1%); diffuse RAS, 52 patients (16%); and RAO, 29 patients (9%). The proportion of the morphological patterns of radial artery injury was not much different between the entire cohort and patients undergoing both pre-PCI and follow-up RAG. As compared with patients without RAS/RAO, patients with RAS/RAO had smaller pre-PCI radial artery diameter) in addition to lower height, smaller body weight, smaller body mass index, and smaller BSA. Multivariable regression analysis identified pre-PCI radial artery diameter, as well as a history of TRA-PCI and BSA as independent predisposing factors of RAS/RAO (Table 4).

**Table 3:** Characteristics of patients undergoing both pre-PCI and follow-up radial artery angiography

	Total (*N* = 323)	RAS/RAO (–) (*n* = 239, 74%)	RAS/RAO ( +)			*p* value for RAS/RAO (–) vs RAS/RAO ( +)
			Total (*n* = 84, 26%)	RAS (*n* = 55, 17%) [focal, 3; diffuse, 52]	RAO (*n* = 29, 9%)	
Age, years	67.3 ± 9.3	66.8 ± 9.0	68.9 ± 10.0	71.0 ± 8.2	64.8 ± 11.9	0.080
Women	58 (18)	34 (14)	24 (29)	15 (27)	9 (31)	0.003
Height, cm	161.6 ± 8.9	162.4 ± 8.7	159.5 ± 9.0	160.2 ± 8.4	158.0 ± 10.2	0.0098
Body weight, kg	65.2 ± 12.1	67.0 ± 12.1	60.0 ± 10.9	60.4 ± 10.3	59.2 ± 11.9	< 0.001
Body mass index, kg/m^2^	24.9 ± 3.5	25.4 ± 3.5	23.5 ± 3.4	23.5 ± 3.6	23.5 ± 3.0	< 0.001
Body surface area, m^2^ (per log 1.1 m^2^ increase)	1.73 ± 0.19	1.76 ± 0.19	1.65 ± 0.18	1.66 ± 0.16	1.63 ± 0.21	< 0.001
History of smoking	174 (54)	130 (54)	44 (52)	27 (49)	17 (59)	0.75
History of PCI	129 (40)	95 (40)	34 (40)	20 (36)	14 (48)	0.91
History of TRA-PCI	59 (18)	49 (21)	10 (12)	10 (18)	0 (0)	0.0794
Hypertension	193 (60)	143 (60)	50 (59)	32 (58)	18 (62)	0.96
Dyslipidemia	181 (56)	136 (57)	45 (54)	24 (44)	21 (72)	0.60
Diabetes	129 (40)	95 (40)	34 (40)	26 (47)	8 (28)	0.91
Acute coronary syndrome	6 (2)	5 (2)	1 (1)	0 (0)	1 (4)	0.60
Procedure time (min)	54.2 ± 38.3	54.5 ± 39.2	53.2 ± 35.7	53.1 ± 32.7	53.3 ± 41.6	0.81
Oral anticoagulants	22 (7)	17 (7)	5 (6)	4 (7)	1 (3)	0.75
Puncture site bleeding requiring prolonged compression	91 (28)	66 (28)	25 (30)	16 (29)	9 (3)	0.63
Pre-PCI radial artery diameter (mm)	3.3 ± 0.6	3.4 ± 0.6	3.0 ± 0.5	2.9 ± 0.4	3.1 ± 0.7	< 0.001

Data are *n* (%) or mean (SD), unless otherwise specified

*PCI* percutaneous coronary intervention, *TRA* transradial approach

**Table 4:** Factors related to radial artery injury in patients undergoing both pre-PCI and follow-up radial artery angiography

	Univariate analysis		Multivariable analysis	
	Odds ratio (95% confidence interval)	*p* value	Odds ratio (95% confidence interval)	*p* value
Age	0.97 (0.94–1.00)	0.080	1 (0.97–1.04)	0.72
Women	2.4 (1.31–4.37)	0.003	1.52 (0.68–3.50)	0.31
Body surface area (per log 1.1 m^2^ increase)	1.62 (1.30–2.05)	< 0.001	1.61 (1.16–2.24)	0.004
Procedure time	1.33 (0.20–10.0)	0.77	0.82 (0.69–9.65)	0.87
Oral anticoagulants	1.18 (0.45–3.68)	0.75	1.20 (0.64–2.30)	0.27
Puncture site bleeding requiring prolonged compression	0.73 (0.30–2.01)	0.51	0.89 (0.51–1.57)	0.58
History of TRA-PCI	1.91 (0.95–4.17)	0.080	2.36 (1.10–5.49)	0.026
Pre-PCI radial artery diameter (per log 1.1 mm increase)	1.55 (1.33–1.82)	< 0.001	1.15 (1.10–1.23)	< 0.001

Height, body weight, and body mass index were omitted from Table 4 due to possible multicollinearity. Abbreviations as in Table 3

## Discussion

The major findings in this study were as follows: (1) radial artery injury including RAS and RAO at 1 year after TRA-PCI was 25% with 3 distinct morphological patterns: focal RAS, diffuse RAS and RAO; (2) not only patients with RAO, significant proportion of patients with RAS required conversion of access site to other sites for repeated PCI; (3) BSA, a history of TRA-PCI, and pre-PCI radial artery diameter were independently related to radial artery injury.

TRA-PCI has spread across the world in the past two decades, with improved patient comfort and outcomes better than the transfemoral approach for PCI [1–3, 5–7, 15–18]. However, injury to the access site artery is known to be an important complication of TRA-PCI. [8–10, 19, 20]. Although Allen's test has been used for the assessment of the risk for ischemic complications after TRA-PCI, previous study reported the limited accuracy of this test [21]. Instead, previous studies have evaluated the incidence of RAO using ultrasound. However, reported incidences varied widely from < 1 to 33%, due to the differences in the methods used for detecting RAO, the sheath size used for PCI, and the timing of assessment [10, 20–25]. Buturak et al. investigated radial artery patency at 6–15 months after TRA-PCI and reported that the incidence of RAO was 19.5% [12]. Lisowska et al. investigated radial artery patency immediately after TRA-PCI and in long-term observation and reported that the 6- to 12-month incidence of RAO was 12.7% [13]. Present study evaluated radial artery injury at 12-month follow-up using RAG, which could definitely detect late-phase RAS as well as RAO. We found that incidence of RAO in the entire study population was 9%, while 17% of the study patients showed RAS. We included RAS as a significant injury to radial artery, because even RAS would be clinically problematic. Indeed, 28 out of 39 patients (72%) with RAS required conversion of access site to other sites at the repeated PCI.

Smaller BSA, a history of TRA-PCI, and smaller pre-PCI radial artery diameter were identified to be related to radial artery injury, which was consistent with a previous study [10]. Presumably, the mechanism of radial artery injury is multifactorial, including damage to endothelial cells of the radial artery, arterial spasm, and thrombus formation [19]. Damage to endothelial cells exerted by repeated sheath insertion may induce tissue necrosis, intimal hyperplasia, and adventitial inflammation [26, 27]. Theoretically, a smaller ratio of the radial artery to the sheath leads to a broader contact area between the sheath and the endothelium of the radial artery, causing more damage to the endothelium of the radial artery. In addition, a smaller radial artery diameter may have an increased risk of disturbing arterial flow at the top of the sheath [8]. Consequent hemostasis may provide the nidus for thrombus formation, leading to thrombotic RAO.

Both pharmacologic and non-pharmacologic strategies including a low ratio of the sheath size to the artery size, the administration of intra-procedural heparin, the maintenance of radial artery patency by ipsilateral ulnar compression during the hemostasis after TRA-PCI have been shown to potentially decrease the risk of radial artery injury, which can be options for high-risk patients [23, 28–30]. Of note, an international survey showed that the incidence of RAO before hospital discharge was not assessed in more than 50% of the patients [9]. Given that radial artery injury is a considerably common complication, radial artery patency should be examined in all patients after TRA-PCI.

Our study is limited by its retrospective single-center observational study. In addition, we did not include patients with severe renal dysfunction because we did not perform RAG on them to avoid the risk of worsening renal function. Furthermore, we performed RAG via the sheath inserted in the brachial artery in a direction contrary to blood flow. Thus, blood flow to the radial artery might have been disturbed by the sheath, leading to the possible underestimation of the actual diameter of the radial artery. However, we used a 5-Fr arterial sheath for both pre-PCI and follow-up diagnostic catheterization to avoid complications of puncture site [14]. Because 5-Fr sheath is seemingly narrow enough to maintain distal arterial flow, we believe that we were able to precisely measure the actual diameter of the radial artery. 4 patients (0.7%) developed pseudo-aneurysm at the brachial puncture site, requiring surgical treatment in this study. Although the incidence rate was lower than that reported in the previous study using 6-Fr sheath [14], brachial artery approach has been less likely used in the current practice.

In conclusion, we evaluated radial artery injury after TRA-PCI by RAG and found that a long-term incidence of radial artery injury after TRA-PCI was considerably high. Thus, we would like to propose that more attention be paid to radial artery injury, especially in patients with smaller body surface area, a history of TRA-PCI, and smaller pre-PCI radial artery diameter.
